# Intraperitoneal Polypropylene Mesh in Clean, Potentially Septic, and Controlled Contamination Fields: An Experimental Rat Study

**DOI:** 10.3390/medicina62050803

**Published:** 2026-04-22

**Authors:** Apostolos Makrantonakis, Ioannis Mantzoros, Orestis Ioannidis, Konstantinos Zapsalis, Elissavet Anestiadou, Styliani Parpoudi, Dimitrios Kyziridis, Ekaterini Klonou, Savvas Simeonidis, Stefanos Bitsianis, Manousos George Pramateftakis, Efstathios Kotidis, Stamatios Angelopoulos

**Affiliations:** 4th Surgical Clinic, School of Medicine, Faculty of Health Science, Aristotle University of Thessaloniki, 541 24 Thessaloniki, Greece; aloucardo@hotmail.com (A.M.); elissavetxatz@gmail.com (E.A.); stellaparpoudi@hotmail.com (S.P.); dkyziridis@gmail.com (D.K.); ekatklon@outlook.com (E.K.); simeonidissavvas@yahoo.com (S.S.); mpramateftakis@hotmail.com (M.G.P.); skotidis@gmail.com (E.K.); saggelopoulos@auth.gr (S.A.)

**Keywords:** intraperitoneal mesh, polypropylene, adhesions, sepsis, contaminated surgical field, anastomosis, ventral/incisional hernia repair, rat model

## Abstract

*Background and Objectives*: Intraperitoneal onlay mesh (IPOM) reduces ventral/incisional hernia recurrence but raises concern for adhesions and infection, particularly when the operative field is not strictly clean. We aimed to determine how contamination severity modulates the peritoneal response to intraperitoneal polypropylene mesh. *Materials and Methods*: In a prospective, randomized, blinded rat study, 60 male Wistar rats were allocated to three groups (*n* = 20/group) and evaluated at postoperative day (POD) 4 and POD 8 (n = 10/timepoint): A, clean mesh placement; B, small-bowel resection with end-to-end anastomosis without spillage (“potentially septic”); and C, mesh placement followed by intraperitoneal inoculation with *Escherichia coli* and *Staphylococcus aureus* (“controlled contamination”). The primary outcome was adhesion severity (Van der Ham scale, 0–3). Secondary outcomes included semi-quantitative histological scores (0–4) for neutrophil infiltration, fibroblast proliferation, neoangiogenesis, and collagen deposition. Prespecified non-parametric analyses were applied. *Results*: All animals completed follow-up; no pre-sacrifice deaths occurred. Adhesion severity showed no statistically significant differences between Groups A and B at either timepoint (mean POD4: 0.3 vs. 0.6; POD8: 0.4 vs. 0.8; *p* > 0.05). In contrast, Group C demonstrated markedly higher adhesion scores (mean POD4: 2.3; POD8: 2.4; both *p* < 0.001 vs. Groups A and B), with a substantially greater proportion of grade 2–3 adhesions. Histological parameters paralleled these findings: at both POD4 and POD8, Group C showed significantly higher neutrophil, fibroblast, neoangiogenesis, and collagen scores compared with Groups A and B (all *p* < 0.001). No statistically significant within-group temporal differences were observed between POD4 and POD8. *Conclusions*: In this experimental model, intraperitoneal polypropylene mesh demonstrated similar early biological response patterns in clean and controlled contamination settings, whereas established intra-abdominal sepsis was associated with a marked escalation of inflammation, fibroproliferation, and adhesion formation. These findings suggest that selective use of synthetic intraperitoneal mesh may be considered when contamination is controlled, while caution is warranted in frankly septic environments.

## 1. Introduction

Ventral and incisional hernia repair has progressively shifted toward prosthetic reinforcement to reduce recurrence, and intraperitoneal onlay mesh (IPOM) has been widely adopted—particularly in minimally invasive surgery—because it offers broad defect coverage and durable reinforcement [1,2,3,4]. However, intraperitoneal placement inherently creates direct mesh–viscera contact, which may provoke adhesions and mesh-related complications, including chronic pain, infection, enterocutaneous fistulae, bowel obstruction, and challenging reoperations [5,6,7,8,9,10,11]. Postoperative adhesions, in particular, remain a major source of long-term morbidity and readmissions after abdominal surgery and constitute a substantial healthcare burden [5,6,10].

Peritoneal healing after surgery reflects a dynamic balance between fibrin deposition, fibrinolysis, and fibroproliferation. Disruption of this balance—especially in the setting of inflammation or infection—promotes organization of fibrinous bridges into mature adhesions via fibroblast recruitment, neoangiogenesis, and collagen deposition [7,8,12,13]. The presence of a foreign body may amplify these pathways by acting as a scaffold for cellular infiltration and bacterial adherence/biofilm formation, thereby sustaining inflammation and stabilizing adhesion bands [13,14]. Accordingly, mesh characteristics (polymer type, filament structure, pore size, density, and barrier coatings) have been targeted to mitigate adhesions, although anti-adhesive layers may degrade over time, and long-term protective effects remain variable [9,15,16,17].

Despite the clinical relevance, the role of synthetic mesh in non-clean fields remains controversial. Observational registry and database studies have reported higher infectious morbidity when mesh is used in clean-contaminated or contaminated ventral hernia repairs—particularly when bowel resection is performed [18,19]. Conversely, other clinical series and experimental work suggest that mesh use may be feasible when contamination is controlled and meticulous technique prevents spillage or diffuse peritonitis, raising the possibility that “potential contamination” may behave biologically closer to a clean field than to frank sepsis [16,20,21,22]. Contemporary hernia guidelines also emphasize tailoring technique and material selection to patient factors, defect characteristics, and contamination status but acknowledge that evidence is heterogeneous and limited in truly septic settings [23].

This apparent inconsistency may reflect a threshold phenomenon: controlled contamination (e.g., a sealed bowel anastomosis without spillage) may not be sufficient to transform the mesh–peritoneum interface into a pro-adhesive, pro-infectious environment, whereas established bacterial peritonitis may fundamentally alter host response and mesh integration. To test this hypothesis under controlled conditions, we conducted a randomized rat study comparing peritoneal responses to intraperitoneal polypropylene mesh in (i) clean conditions, (ii) potentially septic conditions created by bowel resection and end-to-end anastomosis without deliberate contamination, and (iii) frank sepsis induced by intraperitoneal inoculation with *Escherichia coli* and *Staphylococcus aureus*.

## 2. Materials and Methods

### 2.1. Study Design, Animals, Housing, Ethics

A prospective, randomized, controlled animal study was performed in male Wistar rats (5–8 months, 250–500 g). Animals were housed under a 12 h light/dark cycle with standard chow (EL.VI.Z 510) and water ad libitum; a 12 h fast with free water was observed preoperatively. Procedures were performed in the experimental OR (G. Papanikolaou Hospital) under institutional and European guidelines. Animals were randomized (simple random allocation) into three equal groups (n = 20 each): A (control—clean), B (anastomosis—potentially septic), and C (controlled contamination). Each group had two sacrifice timepoints, POD 4 and POD 8 (n = 10/timepoint).

All experimental procedures were approved prior to study initiation by the Veterinary Service of Thessaloniki (Regional Veterinary Authority), under approval number 23962/121 (approved on 10 November 2021). The study was conducted in accordance with Directive 2010/63/EU on the protection of animals used for scientific purposes and applicable national legislation. All procedures adhered to the principles of replacement, reduction, and refinement (3Rs) to minimize animal suffering and optimize experimental design. The study was designed, conducted, and reported in accordance with the ARRIVE 2.0 guidelines for reporting animal research.

### 2.2. Randomization and Blinding

Animals were randomly allocated (1:1:1; n = 20/group) to experimental groups using a computer-generated randomization sequence prior to surgery. Each group was subdivided by timepoint (POD4 and POD8; n = 10/timepoint). Owing to the nature of the surgical interventions, the operating surgeon could not be blinded to group allocation. Group allocation was concealed from outcome assessors. However, macroscopic adhesion scoring and histological assessment were performed by investigators blinded to group assignment. Statistical analyses were conducted using anonymized group codes to minimize assessment bias.

### 2.3. Anesthesia and Operative Technique

The experimental unit was defined as a single animal. General anesthesia was induced and maintained using intraperitoneal ketamine hydrochloride (Imalgene^®^, Merial, Lyon, France) (80 mg/kg) and xylazine hydrochloride (Rompun^®^, Bayer AG, Leverkusen, Germany) (8 mg/kg). Following sterile preparation, a midline laparotomy was performed.

A 2 × 2 cm segment was cut from a sterile, uncoated, nonabsorbable, monofilament polypropylene 30 × 30 cm mesh (Prolene^®^ Mesh 30 × 30 cm, Ethicon Inc., Somerville, NJ, USA), which was used in all experimental groups. The mesh was placed intraperitoneally in direct contact with the parietal peritoneum and fixed using interrupted 4-0 polypropylene (PROLENE^®^, Ethicon Inc., Somerville, NJ, USA) sutures. Before abdominal closure, ciprofloxacin (10 mg/kg) (Ciproxin^®^, Bayer AG, Leverkusen, Germany) was administered as a diluted solution in 2 mL sterile normal saline and instilled intraperitoneally as prophylaxis prior to abdominal closure.

Group-specific steps:

- Group A (control): Intraperitoneal mesh placement only.
- Group B (anastomosis/potentially septic): A 2 cm segmental small-bowel resection was performed 5 cm proximal to the ileocecal valve, followed by end-to-end anastomosis using interrupted 4-0 polyglactin 910 sutures (VICRYL^®^, Ethicon Inc., Somerville, NJ, USA). Mesh placement was then completed as described above.
- Group C (controlled contamination): Mesh placement was followed by intraperitoneal inoculation with a standardized polymicrobial suspension consisting of *Escherichia coli* (approximately 1 × 10^6^ CFU per animal) and Staphylococcus aureus (approximately 1 × 10^6^ CFU per animal). Bacterial strains were cultured overnight on blood agar plates at 37 °C, and colonies were transferred into sterile saline. Suspensions were adjusted to the desired concentration using optical density calibration (0.5 McFarland standard) and serial dilution techniques. The final inoculum was prepared to deliver the target CFU count per organism in a total intraperitoneal volume of 200 μL, administered immediately after mesh fixation. Bacterial concentration was verified prior to inoculation using standard colony-forming unit (CFU) quantification by serial dilution and plate counting.

Peritonitis severity was not graded using a predefined, validated scoring system or quantitative microbiological cultures. Instead, induction of a septic intra-abdominal environment was assessed indirectly through macroscopic intra-abdominal findings at relaparotomy and through the prespecified biological study endpoints, including adhesion severity grading, presence of abscess-associated adhesions, and semi-quantitative histopathological evaluation of inflammatory and fibroproliferative response, including neutrophil infiltration, fibroblast proliferation, neoangiogenesis, and collagen deposition.

**Figure 1:**
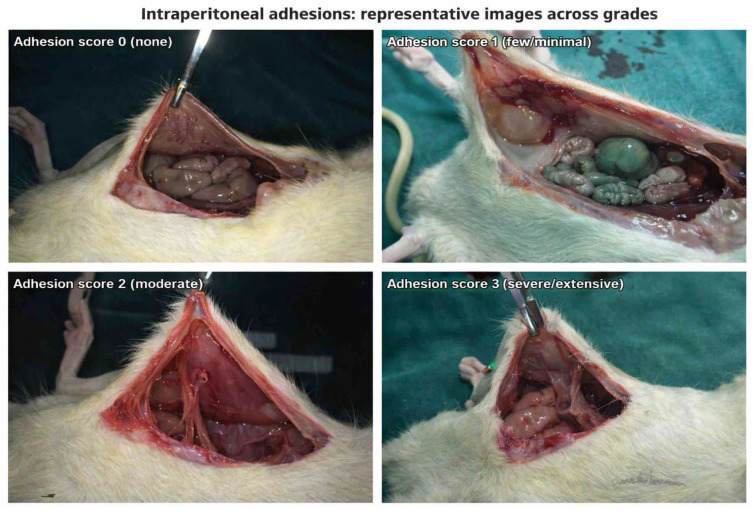
Score 0 (top-left): no adhesions; smooth peritoneal surfaces with freely mobile bowel loops. Score 1 (top-right): few, thin, filmy adhesions that separate easily with gentle traction. Score 2 (bottom-left): localized, thicker vascularized bands. Score 3 (bottom-right): extensive, dense vascular adhesions between the abdominal wall and viscera.

**Figure 2:**
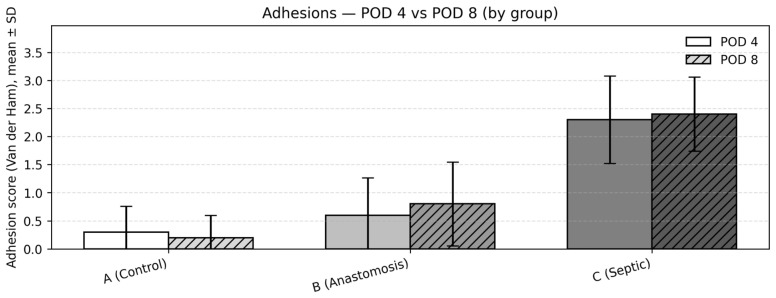
Adhesions—POD 4 vs. POD 8 (by group): Grouped bar chart showing the mean ± SD of the Van der Ham adhesion score (0–3) for each group: A (clean), B (potentially septic—anastomosis), and C (controlled contamination). Bars are grayscale by group (A, light grey; B, medium grey; C, dark grey); within each group, POD 4 is the lighter, unhatched bar and POD 8 the darker, diagonally hatched bar. Error bars denote the standard deviation. As observed in the study, adhesion burden is significantly higher in Group C compared with Groups A and B, while Groups A and B do not differ significantly; no material time effect between POD 4 and POD 8 was detected. Abbreviations: POD, postoperative day. The distribution of adhesion scores within each group is additionally illustrated using a grouped bar representation to allow for visual comparison of ordinal score distributions.

Intraoperative photographs can be seen in Figure 1 and Figure 2.

In the present study, the term controlled contamination refers to experimental bacterial inoculation designed to induce intra-abdominal sepsis, whereas potential contamination refers to bowel resection with anastomosis performed without spillage.

### 2.4. Outcomes, Sampling, and Definitions

At POD 4 or POD 8, re-laparotomy and sacrifice were performed. Semi-quantitative histological scoring was performed using predefined criteria adapted from previously published experimental mesh and peritoneal inflammation models. The scoring systems for neutrophil infiltration, fibroblast proliferation, neoangiogenesis, and collagen deposition (each graded on a 0–4 scale) are consistent with validated frameworks described in experimental studies of mesh-related tissue reaction [24]. All histological assessments were conducted by a blinded investigator using coded slides to ensure unbiased evaluation.

Primary endpoint: intraperitoneal adhesions graded on the Van der Ham scale [25] (0 = none; 1 = few/minimal; 2 = moderate; 3 = severe/extensive, often with abscess). 

Secondary histology: semi-quantitative 0–4 scoring for neutrophil infiltration, fibroblasts, neoangiogenesis, and collagen deposition (0 = absent; 1 = occasional; 2 = slightly increased; 3 = frequent; 4 = confluent). Group and timepoint distributions are displayed in tables/figures (see below). This is based on previous studies that also used this scale [26].

### 2.5. Statistics and Sample Size Calculation

All statistical analyses were performed using non-parametric methods due to the violation of normality assumptions and the ordinal nature of the primary outcome variables. Normality of continuous and ordinal variables was assessed using the Shapiro–Wilk test, which demonstrated a non-normal distribution across groups and timepoints. In addition, adhesion grades and semi-quantitative histological scores represent ordinal variables; therefore, parametric statistical testing was considered inappropriate for final inferential analysis. Statistical analyses were performed using Jamovi software (version 2.7) with additional verification of effect size metrics using standard non-parametric statistical formulas.

For comparisons among more than two independent groups, the Kruskal–Wallis test was used. When a statistically significant overall difference was detected, post hoc pairwise comparisons were performed using the Dwass–Steel–Critchlow–Fligner (DSCF) test, which appropriately adjusts for multiple comparisons while maintaining statistical power in non-parametric datasets.

For comparisons between two independent groups, including comparisons between postoperative timepoints within the same experimental group, the Mann–Whitney U test was used.

Results are presented as mean ± standard deviation for descriptive purposes to facilitate comparison with previous experimental studies, while statistical inference was based exclusively on rank-based non-parametric tests appropriate for ordinal outcomes. A two-tailed *p*-value < 0.05 was considered statistically significant.

Sample size was determined based on feasibility and adherence to ethical principles for animal research (3Rs) while aiming to detect biologically meaningful differences in the primary endpoint (Van der Ham adhesion score). A power calculation was conducted using a two-sided significance level (α) of 0.05 and statistical power of 80%. Based on variability reported in previous experimental adhesion studies and preliminary observations from similar models, the standard deviation of adhesion scores was assumed to be approximately 0.7–0.8. Under these assumptions, detection of a difference of at least one point in the adhesion score between groups required approximately 9–10 animals per group using a two-sample comparison framework.

Given the three-group design, overall group differences were evaluated using the Kruskal–Wallis test followed by Dwass–Steel–Critchlow–Fligner post hoc comparisons, which account for multiple pairwise comparisons in non-parametric datasets. Because adhesion grades and histological parameters represent ordinal outcomes and normality assumptions were violated, non-parametric statistical methods were used for the final inferential analyses.

Effect sizes were calculated to complement statistical significance testing. For Kruskal–Wallis analyses, epsilon-squared (ε^2^) was used as a measure of effect size to estimate the proportion of variance explained by group differences. For pairwise comparisons using the Mann–Whitney U test, rank-biserial correlation coefficients were calculated to quantify the magnitude of differences between groups. Effect sizes were interpreted according to commonly accepted thresholds for non-parametric analyses. For epsilon-squared values, thresholds of approximately 0.01, 0.08, and 0.26 were considered indicative of small, moderate, and large effects, respectively.

The sample size was therefore considered sufficient to detect biologically meaningful differences between septic and non-septic conditions. However, the study was not specifically powered to detect small differences between the clean and potentially septic groups. In addition, the study design included two postoperative assessment timepoints (POD4 and POD8); however, statistical comparisons across timepoints were considered exploratory, and no additional multiplicity adjustment beyond the non-parametric post hoc testing was applied.

Graphical representations of score distributions were used to visually illustrate group differences in ordinal outcome variables.

## 3. Results

### 3.1. Cohort

All 60 Wistar rats completed follow-up to planned sacrifice; no pre-sacrifice deaths occurred. Consistent with effective induction of a septic intra-abdominal environment, animals in the “controlled contamination” group demonstrated uniformly higher adhesion grades, including abscess-associated adhesions, together with markedly increased inflammatory and fibroproliferative histological scores across both postoperative timepoints. Because the primary outcome variables (adhesion grades and semi-quantitative histological scores) were ordinal and non-normally distributed, all intergroup comparisons were performed using non-parametric statistical methods. In addition to *p*-values, effect size estimates were calculated to determine the magnitude of group differences. For the Kruskal–Wallis analyses, epsilon-squared values indicated a large effect size for the influence of contamination status on adhesion severity and histological inflammatory responses.

### 3.2. Adhesions

Adhesions were evaluated using the Van der Ham scale (0–3). At both POD4 and POD8, adhesion scores were low in Groups 1 and 2 and markedly higher in Group 3, demonstrating a consistent rightward shift in adhesion severity distribution under septic conditions (Figure 1 and Figure 2).

Between-group comparisons (POD4)

Kruskal–Wallis analysis demonstrated a statistically significant difference in adhesion scores between the groups (*p* < 0.001). The corresponding epsilon-squared effect size indicated a large effect of contamination status on adhesion severity, suggesting that group allocation explained a substantial proportion of the variability in adhesion scores.

Mean (SD) adhesion scores were 0.3 (0.48) in Group 1, 0.6 (0.70) in Group 2, and 2.3 (0.82) in Group 3. Post hoc pairwise comparisons using the Dwass–Steel–Critchlow–Fligner test showed no statistically significant difference between Groups 1 and 2 (*p* = 1.000), whereas Group 3 demonstrated significantly higher adhesion scores compared with both Group 1 and Group 2 (both *p* < 0.001).

Between-group comparisons (POD8)

Kruskal–Wallis analysis again demonstrated a statistically significant difference between groups (*p* < 0.001). Mean (SD) adhesion scores were 0.4 (0.52) in Group 1, 0.8 (0.79) in Group 2, and 2.4 (0.70) in Group 3. Post hoc pairwise comparisons showed no statistically significant difference between Groups 1 and 2 (*p* = 0.594), while adhesion scores remained significantly higher in Group 3 compared with Groups 1 and 2 (both *p* < 0.001).

Change over time within groups (POD4 → POD8)

Comparisons between postoperative timepoints within each group were performed using the Mann–Whitney U test. No statistically significant differences were observed between POD4 and POD8 adhesion scores within any group:

- Group 1: 0.3 → 0.4 (*p* = 0.660);
- Group 2: 0.6 → 0.8 (*p* = 0.556);
- Group 3: 2.3 → 2.4 (*p* = 0.773).

### 3.3. Histology

Across all four histological parameters, controlled contamination conditions (Group 3) were consistently associated with markedly increased inflammatory and fibroproliferative responses at both timepoints, with large effect sizes observed in the corresponding non-parametric comparisons. Clean conditions and anastomosis without overt contamination (Groups 1 and 2) resulted in similarly low histological scores, with no statistically significant differences observed between them. Temporal analysis showed no statistically significant changes between POD4 and POD8, suggesting that histopathological changes were established early and remained relatively stable during the first postoperative week.

**Figure 3:**
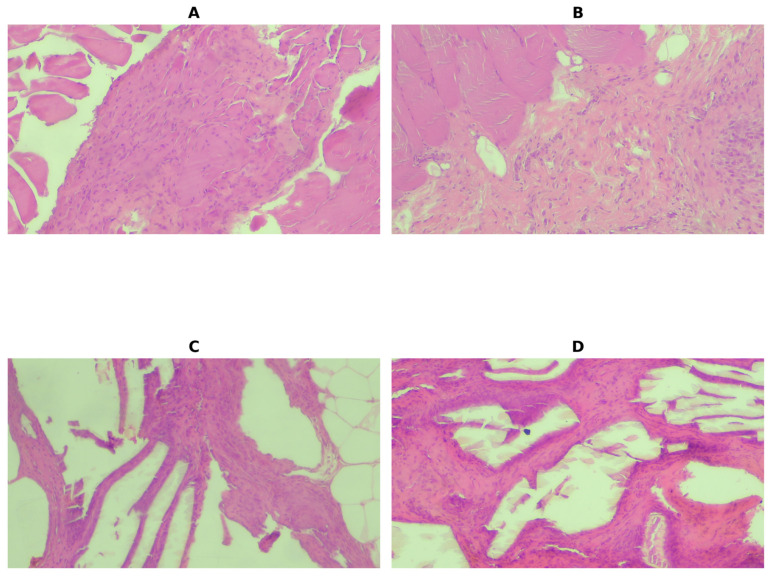
Representative histopathological findings at the mesh–tissue interface (H&E staining). (Objective 10×- Total magnification 100×): (**A**) mild fibroblast proliferation and sparse inflammatory cell infiltration, consistent with a low-grade tissue response observed in clean conditions (Group A); (**B**) early collagen deposition with loosely organized fibers and minimal cellularity, indicating limited fibrotic remodeling (Group B); (**C**) increased fibroblast density and neoangiogenesis, reflecting an active fibroproliferative response typical of septic conditions (Group C); (**D**) dense collagen deposition with marked cellular infiltration and disorganized tissue architecture, consistent with advanced inflammatory and fibrotic reactions (Group C).

Some representative histopathological findings at the mesh–tissue interface after H&E staining can be found in Figure 3.

### 3.4. Neutrophil Infiltration

Between-group comparisons (POD4)

**Figure 4:**
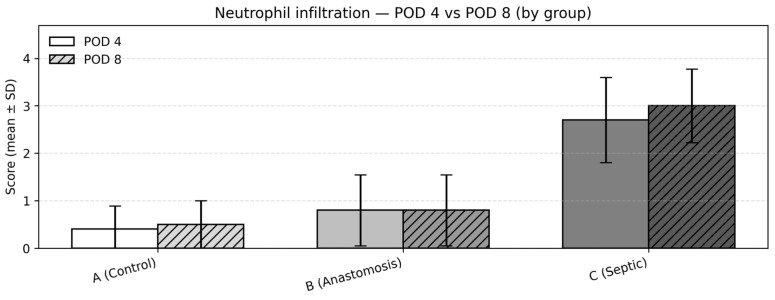
Neutrophil infiltration—POD 4 vs. POD 8: mean ± SD of the semi-quantitative neutrophil score by group.

At POD4, neutrophil infiltration scores differed significantly among the groups (Kruskal–Wallis test, *p* < 0.001). Mean (SD) scores were 0.4 (0.52) in Group 1, 0.8 (0.79) in Group 2, and 2.7 (0.95) in Group 3. Post hoc pairwise comparisons using the Dwass–Steel–Critchlow–Fligner test showed no statistically significant difference between Groups 1 and 2, whereas Group 3 demonstrated significantly higher neutrophil infiltration compared with both Groups 1 and 2 (both *p* < 0.001) (Figure 4).

Between-group comparisons (POD8)

At POD8, this pattern persisted, with a statistically significant difference observed among the groups (Kruskal–Wallis test, *p* < 0.001). Mean (SD) neutrophil scores were 0.5 (0.53) in Group 1, 0.8 (0.79) in Group 2, and 3.0 (0.82) in Group 3. Post hoc pairwise comparisons again confirmed significantly higher neutrophil infiltration in Group 3 compared with Groups 1 and 2, with no statistically significant difference observed between Groups 1 and 2 (Figure 4).

Change over time within groups (POD4 → POD8)

Comparisons between POD4 and POD8 within each group were performed using the Mann–Whitney U test. No statistically significant differences were observed between timepoints within any group (Group 1: *p* = 0.673; Group 2: *p* = 1.000; Group 3: *p* = 0.458), suggesting relatively stable inflammatory activity during the first postoperative week.

### 3.5. Fibroblast Proliferation

Between-group comparisons (POD4)

**Figure 5:**
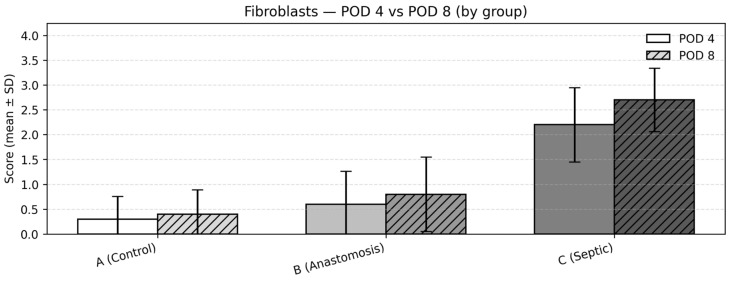
Fibroblasts—POD 4 vs. POD 8: mean ± SD of fibroblast proliferation scores.

At POD4, fibrosis scores differed significantly among the groups (Kruskal–Wallis test, *p* < 0.001). Mean (SD) scores were 0.3 (0.48) in Group 1, 0.6 (0.70) in Group 2, and 2.2 (0.79) in Group 3. Post hoc pairwise comparisons using the Dwass–Steel–Critchlow–Fligner test demonstrated significantly higher fibrosis scores in Group 3 compared with both Groups 1 and 2 (both *p* < 0.001), while no statistically significant difference was observed between Groups 1 and 2 (Figure 5).

Between-group comparisons (POD8)

At POD8, fibrosis scores showed a numerical increase while maintaining the same intergroup distribution pattern (Kruskal–Wallis test, *p* < 0.001). Mean (SD) values were 0.4 (0.52) in Group 1, 0.8 (0.79) in Group 2, and 2.7 (0.67) in Group 3. Group 3 remained significantly higher than both Groups 1 and 2 (both *p* < 0.001), with no statistically significant difference observed between Groups 1 and 2 (Figure 5).

Change over time within groups (POD4 → POD8)

Within-group comparisons between POD4 and POD8 were performed using the Mann–Whitney U test. No statistically significant differences were observed between timepoints within any group (Group 1: *p* = 0.660; Group 2: *p* = 0.556; Group 3: *p* = 0.145).

### 3.6. Neoangiogenesis

Between-group comparisons (POD4)

**Figure 6:**
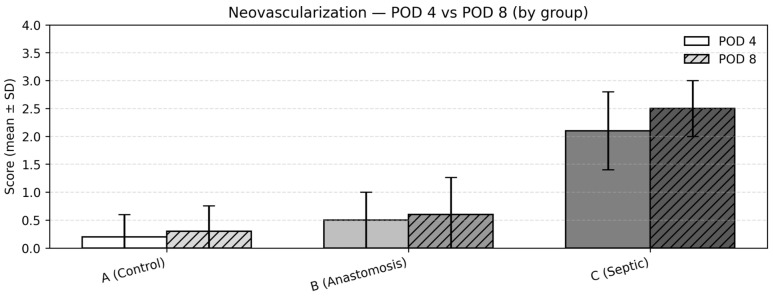
Neovascularization—POD 4 vs. POD 8: mean ± SD of neovascularization scores by group.

At POD4, neoangiogenesis scores differed significantly among the groups (Kruskal–Wallis test, *p* < 0.001). Mean (SD) scores were 0.2 (0.42) in Group 1, 0.5 (0.53) in Group 2, and 2.1 (0.74) in Group 3. Post hoc pairwise comparisons using the Dwass–Steel–Critchlow–Fligner test demonstrated significantly increased neoangiogenesis in Group 3 compared with both Groups 1 and 2 (both *p* < 0.001), while no statistically significant difference was observed between Groups 1 and 2 (Figure 6).

Between-group comparisons (POD8)

At POD8, differences remained pronounced (Kruskal–Wallis test, *p* < 0.001). Mean (SD) scores were 0.3 (0.48) in Group 1, 0.6 (0.70) in Group 2, and 2.5 (0.53) in Group 3. Group 3 again demonstrated significantly higher vascular proliferation compared with both Groups 1 and 2 (both *p* < 0.001), with no statistically significant difference observed between Groups 1 and 2 (Figure 6).

Change over time within groups (POD4 → POD8)

Within-group comparisons between POD4 and POD8 were performed using the Mann–Whitney U test. No statistically significant differences were observed between timepoints within any group (Group 1: *p* = 0.628; Group 2: *p* = 0.722; Group 3: *p* = 0.180).

### 3.7. Collagen Deposition

Between-group comparisons (POD4)

**Figure 7:**
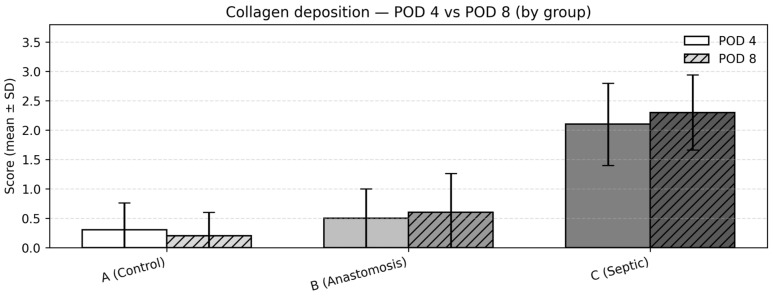
Collagen deposition—POD 4 vs. POD 8: mean ± SD of collagen deposition scores.

At POD4, collagen deposition scores differed significantly among the groups (Kruskal–Wallis test, *p* < 0.001). Mean (SD) scores were 0.3 (0.48) in Group 1, 0.5 (0.53) in Group 2, and 2.1 (0.74) in Group 3. Post hoc pairwise comparisons using the Dwass–Steel–Critchlow–Fligner test demonstrated significantly greater collagen deposition in Group 3 compared with both Groups 1 and 2 (both *p* < 0.001), while no statistically significant difference was observed between Groups 1 and 2 (Figure 7).

Between-group comparisons (POD8)

At POD8, this intergroup pattern persisted (Kruskal–Wallis test, *p* < 0.001). Mean (SD) collagen scores were 0.2 (0.42) in Group 1, 0.6 (0.70) in Group 2, and 2.3 (0.67) in Group 3. Post hoc pairwise comparisons confirmed continued significantly higher collagen deposition in Group 3 compared with both Groups 1 and 2 (both *p* < 0.001), with no statistically significant difference observed between Groups 1 and 2 (Figure 7).

Change over time within groups (POD4 → POD8)

Within-group comparisons between POD4 and POD8 were performed using the Mann–Whitney U test. No statistically significant differences were observed between timepoints within any group (Group 1: *p* = 0.628; Group 2: *p* = 0.722; Group 3: *p* = 0.535).

The full semi-quantitative distribution of histological scores across groups and timepoints is summarized in Table 1 to facilitate direct comparison.

## 4. Discussion

This controlled rat model directly interrogates how the degree of contamination modulates the peritoneal response to standardized intraperitoneal uncoated monofilament polypropylene mesh. Three consistent signals emerge across clinical, histologic, and adhesion endpoints. First, a clean abdomen exposed to a 2 × 2 cm polypropylene implant shows only low-grade, predominantly self-limited inflammation: neutrophils and fibroblasts are absent or occasional in most animals, neovascularization is sparse, collagen deposition remains modest, and adhesions are few to moderate. Second, when a small-bowel resection with end-to-end anastomosis is performed—our “potentially septic” model without deliberate spillage—the adhesion and histological response profiles showed no statistically significant differences compared with clean controls at both POD 4 and POD 8, although formal equivalence was not tested and small intergroup differences cannot be excluded. Third, with overt sepsis triggered by intraperitoneal inoculation of *E. coli* and *S. aureus*, the response is transformed: inflammatory and fibroproliferative indices rise across the board, collagen accumulation advances, and adhesions become more frequent and more severe, including grade-3 formations often accompanied by abscess, with highly significant differences versus both the clean and anastomosis groups (*p* < 0.001 for most pairwise contrasts). These patterns are robust to time, as no within-group differences were detected between POD 4 and POD 8 for any measured variable, underscoring that the early postoperative window already encodes the direction of the mesh–peritoneum interaction in each contamination setting. The uniform increase in adhesion severity, including abscess-associated adhesions, and the parallel escalation of inflammatory and fibroproliferative histological markers in the “controlled contamination” group further support the presence of a clearly distinct septic intra-abdominal biological phenotype compared with controlled contamination and clean conditions.

The absence of detectable divergence between clean and potentially septic settings warrants emphasis. In both arms, neutrophil infiltration, fibroblast presence, angiogenesis, and collagen deposition largely clustered at score 0–1 at POD 4 and remained low at POD 8, with no significant between-group differences across these domains (all *p* > 0.05). Adhesion frequencies and severities by the Van der Hamm scale also overlapped between these two groups (*p* > 0.05), suggesting that the mere presence of an anastomosis, in the absence of bacterial peritonitis, is not enough to convert a polypropylene mesh interface into a pro-adhesive niche in this model. This finding aligns with the intuitive clinical notion that controlled contamination (i.e., no spillage, hemostasis, and meticulous technique) is biologically closer to “clean” than to “septic,” a concept increasingly emphasized in contemporary guideline documents and expert consensus statements [17,18,27]. It also provides experimental ballast to practice patterns wherein surgeons occasionally proceed with synthetic mesh when contamination risk is theoretical rather than realized, provided rigorous intraoperative control is maintained [17,18].

By contrast, in the septic arm, distributions shift decisively toward higher scores across all histologic axes. Neutrophils frequently reach 2–4, fibroblasts and neovascularization often sit at 2–3, and collagen deposition extends beyond 2 in a notable subset. The adhesion profile mirrors this histologic escalation: grade-3 adhesions—extensive and often abscess-associated—appear predominantly in septic animals, driving significant pairwise differences versus both clean and anastomosis groups (commonly *p* < 0.001). In the summarized tables, categories that are virtually unpopulated in clean/potentially septic animals (e.g., neutrophil scores 3–4, fibroblast 3–4) are well represented only in the septic cohort, reinforcing a threshold phenomenon in which bacterial contamination is the principal determinant of adverse mesh–peritoneum dynamics, rather than the anastomosis per se.

Numerous experimental trials have consistently shown that mesh material properties significantly influence adhesion formation and peritoneal inflammatory response. Perko et al. demonstrated variable adhesion burden across different surgical mesh constructs in a rat experimental model, underscoring the material-dependent component of peritoneal healing dynamics [28]. Similarly, Vrijland et al. reported that intraperitoneal polypropylene mesh repair of incisional hernia is not associated with enterocutaneous fistula under conditions of direct visceral contact [29]. In addition, comparative experimental studies have further shown that surface modifications, pore structure, and absorbable coatings may attenuate adhesion formation compared with uncoated polypropylene constructs [27,30]. Reviews of mesh–tissue interaction biology have also emphasized the role of material composition and structural properties in modulating inflammatory and fibroproliferative responses [31].

Taken together, these findings highlight that adhesion formation is influenced not only by contamination severity but also by intrinsic mesh properties. The present study therefore focused specifically on a single standardized uncoated monofilament polypropylene mesh construct in order to isolate the effect of graded intra-abdominal contamination on the early mesh–peritoneum biological response. Consequently, the observations should be interpreted as reflecting the behavior of this specific mesh construct rather than representing all mesh materials used in abdominal wall reconstruction.

Mechanistically, these data reconcile classic models of peritoneal healing and adhesiogenesis with the specific context of intraperitoneal mesh. Following serosal injury, fibrin deposition and mesothelial disruption normally resolve via fibrinolysis; infection prolongs the inflammatory phase, disrupts fibrinolytic balance, and fosters fibroblast recruitment, neovascularization, and collagen maturation into organized adhesions [12,13]. When a foreign body—polypropylene filaments—sits at the injured interface, it can act as a scaffold for cellular infiltration and biofilm formation, augmenting the fibroproliferative program and stabilizing adhesions [15,28,29]. Our septic cohort shows exactly that convergence: an intensified inflammatory milieu (high neutrophils), sustained fibroblast activity and angiogenesis, and greater collagen accrual, all of which culminate in more frequent and severe adhesions. Conversely, in clean and potentially septic settings where bacterial load is controlled, the polypropylene mesh does not, on its own, push the system beyond a low-grade equilibrium—at least within the early postoperative window interrogated here [8,32].

The experimental literature surveyed in the dissertation provides important external context. Multiple studies have documented that material choice, porosity, and coatings influence tissue integration and adhesions, with some coated meshes reducing visceral adhesions versus bare polypropylene in intraperitoneal placement [15,21,22]. At the same time, coating degradation and variable long-term performance complicate blanket recommendations [16]. In contaminated models and peritonitis, certain mesh types perform differently, and recent work has interrogated how mesh characteristics interact with infection to shape outcomes [21,28]. Our study used a single, uncoated polypropylene mesh to isolate the contamination variable; the observed step-up in adverse biology under sepsis suggests that when bacteria are present, even a standard polypropylene interface can become a nidus for exuberant adhesiogenesis and infectious sequelae—an observation concordant with clinical cautions surrounding synthetic mesh in frank contamination [16,18,20].

Clinically, the heterogeneous human data on mesh use in clean-contaminated and contaminated fields have fueled debate. Large registry and database analyses associate mesh with higher wound complications and infections in cases involving bowel resection or contamination [18,19], findings that have been reinforced by more recent large-scale outcomes studies in emergency and high-risk ventral hernia repair [30,33]. Conversely, selected series—including prophylactic mesh in high-risk laparotomies or even peritonitis—report acceptable safety when infection control and operative discipline are optimized, sometimes with coated meshes, and suggest downstream hernia reduction benefits [17,20]. The present results help stratify those realities with respect to the specific polypropylene mesh construct evaluated in this experimental model: when contamination is merely potential and controlled, polypropylene mesh behaves like in clean surgery within the early postoperative window; when peritonitis is established, biology turns decisively unfavorable. These observations should therefore be interpreted as reflecting the early host response to this specific mesh construct rather than representing all mesh platforms used in clinical practice. In practical terms, these data support a nuanced algorithm consistent with contemporary guideline recommendations [27]: in the absence of bacterial spillage or ongoing peritonitis—and with meticulous technique and antibiotic stewardship—synthetic intraperitoneal mesh may be reasonable; in the presence of frank sepsis, the balance of evidence favors avoiding synthetic intraperitoneal placement, considering biologic or specific coated options, or deferring reconstruction until the septic process is resolved [20,22].

Several strengths lend credibility to these conclusions. The study used random allocation to three clinically meaningful conditions: a standardized operative technique (midline laparotomy, consistent mesh size, and fixation with 4-0 Prolene and intraperitoneal ciprofloxacin) and prespecified endpoints spanning clinical adhesions (Van der Hamm) and a semi-quantitative histologic panel (neutrophils, fibroblasts, neoangiogenesis, and collagen). The dual timepoints at POD 4 and POD 8 bracket the acute inflammatory window, capturing both immediate and early-remodeling phases; the lack of within-group temporal differences (all *p* > 0.05) supports the stability of the observed between-group contrasts. The statistical approach was based on non-parametric methods appropriate for ordinal outcomes, including Kruskal–Wallis testing with post hoc pairwise comparisons and Mann–Whitney U tests for between-timepoint analyses, thereby avoiding parametric assumptions.

From a clinical perspective, findings of our study suggest that the biological behavior of intraperitoneal uncoated monofilament polypropylene mesh, as evaluated in this experimental model, is strongly affected by the degree of contamination rather than by the mere presence of a potentially contaminated surgical maneuver such as bowel resection and anastomosis without spillage. In controlled contamination settings, the peritoneal response during the early postoperative period examined in this model appears comparable to that observed in clean conditions, supporting the concept that synthetic intraperitoneal mesh may be considered in carefully selected cases when strict contamination control and meticulous surgical technique are achieved. These observations, however, reflect early host inflammatory and fibroproliferative responses and should not be interpreted as evidence of long-term mesh safety or performance. In contrast, in the presence of established intra-abdominal sepsis, the marked increase in inflammatory response, fibrosis, and adhesion formation observed in this study during the early postoperative phase supports avoidance of intraperitoneal polypropylene placement or consideration of alternative reconstructive strategies. These findings may help inform intraoperative decision-making regarding early biological risk in contaminated settings, although confirmation in studies with longer follow-up is required.

The routine use of perioperative antibiotic coverage in experimental septic mesh models reflects clinical practice but may attenuate microbiological contrasts between groups. A similar approach has been reported by Parpoudi et al. [34,35], where all animals received standardized intraperitoneal ciprofloxacin while septic conditions were induced through bacterial inoculation, allowing for consistent evaluation of host inflammatory and tissue responses under controlled septic conditions. In that experimental framework, biological outcomes such as adhesion severity, histological inflammatory activity, and cytokine response were interpreted primarily as indicators of host response modulation rather than direct bactericidal effects, despite only modest or non-significant differences in culture positivity between groups.

The follow-up period of POD 4 and POD 8 primarily captures early inflammatory and early fibroproliferative phases of peritoneal healing. While these timepoints are critical for adhesion initiation and early mesh–tissue interaction, they do not allow evaluation of adhesion maturation, chronic remodeling, long-term mesh integration, or late mesh-related complications. Therefore, the present findings regarding biological behavior under potentially septic conditions should be interpreted as reflecting an early postoperative response rather than long-term safety.

Similarly, in the present study, the primary signal should be interpreted mainly as a host inflammatory and fibroproliferative response to mesh placement under standardized antimicrobial coverage, rather than as a direct reflection of bacterial burden. While this approach improves internal model stability and translational relevance to perioperative surgical practice, it may reduce microbiological contrasts and should be considered when interpreting contamination threshold effects.

### Limitations and Future Perspectives

Important limitations also temper extrapolation. First, only one polymer (polypropylene), one geometry (2 × 2 cm), and one fixation method were assessed; mesh construction (filament type and pore size), anti-adhesive barriers, and fixation strategies can each influence adhesiogenesis and bacterial interactions, so results may differ with other constructs [29,36,37]. Second, although the inoculum concentration was standardized using CFU quantification, detailed dose–response characterization was not performed. Third, follow-up ended at POD 8, precluding insight into late remodeling, adhesion maturation, mesh encapsulation, shrinkage, and potential fistulization; some coated meshes, for example, show favorable early but not sustained advantages as barriers degrade [16]. Fourth, anesthetic and antibiotic protocols may differ from contemporary human practice and could have modulated inflammatory dynamics. Species differences—wall thickness, peritoneal clearance, immune kinetics—limit linear translation from rats to humans, though comparative work suggests reasonable qualitative parallels between rat and human histology in hernia models [34]. In addition, regarding antibiotic administration, all experimental groups, including the septic cohort, received intraperitoneal ciprofloxacin prior to abdominal closure. Although administered uniformly to ensure animal welfare and reduce excessive mortality, antibiotic use may have attenuated bacterial load and modulated the magnitude of the inflammatory response. Therefore, the observed differences likely reflect mesh behavior under controlled contamination rather than untreated fulminant sepsis. This factor should be considered when interpreting translational implications. However, one should also highlight that the inclusion of standardized antimicrobial therapy may more closely approximate contemporary clinical management of contaminated abdominal surgery, thereby enhancing the translational context of the findings despite potential attenuation of absolute infection severity.

Another limitation of the present study relates to sample size considerations. Although an a priori sample size estimation was performed based on the primary endpoint of adhesion severity, the relatively small subgroup size may limit the ability to detect small intergroup differences, particularly between the clean and potentially septic groups. The study was powered primarily to detect biologically meaningful differences between septic and non-septic conditions rather than subtle differences between the two lower-contamination groups. Consequently, the absence of statistically significant differences between the clean and potentially septic groups should be interpreted with caution, and the possibility of type II error cannot be completely excluded. Furthermore, the power calculation was based on a two-group approximation, as standard sample size formulas for non-parametric three-group ordinal comparisons are not well established for experimental animal models. Furthermore, although effect size metrics were reported to better quantify the magnitude of group differences, the experimental design was primarily intended to detect biologically meaningful contrasts between septic and non-septic conditions rather than subtle differences between the lower-contamination groups.

Moreover, the present study evaluated only a single mesh material (uncoated monofilament polypropylene), which necessarily limits extrapolation to other synthetic, composite, or biologic mesh types. Mesh characteristics—including filament structure, pore size, density, and barrier coatings—may significantly influence inflammatory response, bacterial interaction, and adhesion formation under contaminated conditions. The use of a single standardized construct in this study was intended to isolate contamination-driven biological effects. However, validation across alternative mesh platforms is required before broader material-specific conclusions can be drawn. Although histological scoring was based on established semi-quantitative frameworks adapted from experimental mesh models [24], we did not perform formal interobserver reliability testing. Evaluation was conducted by a single blinded investigator, and future studies incorporating independent observers and reliability metrics would further enhance reproducibility. Last but not least, an additional limitation of the present study is the absence of a combined model including both bowel resection/anastomosis and established intra-abdominal sepsis, as well as the lack of longer-term follow-up beyond 8 postoperative days. Although such a design could provide further insight into long-term mesh viability, anastomotic healing, and late intra-abdominal evolution under septic conditions, this was not feasible within the current experimental framework due to practical and resource-related constraints inherent to complex animal experimental models. It should be noted that these timepoints were selected to capture peak inflammatory and early fibroproliferative activity; however, late remodeling and chronic adhesion maturation require longer observational periods and remain to be investigated.

The absence of quantitative microbiological verification, including colony-forming unit characterization and detailed culture analysis, may limit full reproducibility and may reduce the ability to precisely define the biological threshold between controlled contamination and established sepsis. The experimental framework was designed to evaluate local peritoneal and mesh-related inflammatory responses under graded contamination conditions rather than systemic septic physiology. Additionally, the incorporation of comprehensive systemic biomarker profiling was limited by practical and resource-related constraints inherent to complex animal experimental models. Future investigations integrating quantitative microbiological validation and systemic inflammatory assessment would allow for more complete biological characterization of contamination severity. Future studies incorporating standardized microbiological quantification would further strengthen the mechanistic interpretation of contamination threshold effects. Furthermore, universal antibiotic administration reflects clinical perioperative practice but may attenuate microbiological contrasts and reduce the magnitude of septic inflammatory response. Therefore, the principal biological signal observed in the present study should be interpreted primarily as host inflammatory and tissue response modulation rather than a direct antibacterial effect, and conclusions regarding bacterial clearance should be made cautiously. Even with these caveats, several implications are actionable. For surgeons contemplating intraperitoneal mesh when the field is not strictly clean, the current data argue that the nature of the contamination matters more than the label: an anastomosis executed without spillage did not behave like peritonitis. This supports individualized decision-making in oncologic or complex abdominal reconstructions where delaying mesh could incur other risks. Conversely, when sepsis is established, these findings and the broader literature converge on caution: the combination of foreign material and bacteria prolongs inflammation, recruits fibroblasts and neovessels, accelerates collagen deposition, and stabilizes adhesions—an unfavorable substrate for safe long-term intraperitoneal prostheses [14,19,38]. Selecting a non-intraperitoneal plane (when feasible), deferring the repair, or using specific biologic/coated constructs within carefully controlled protocols may mitigate risk but require confirmation in longer-horizon models and trials, including recent systematic reviews and meta-analyses comparing biologic and synthetic meshes [24,31,39,40,41,42]. Additionally, the present study was not designed or powered to formally test biological equivalence between clean and potentially septic conditions; therefore, the absence of statistically significant differences should not be interpreted as proof of equivalence.

Finally, the study spotlights avenues for future research. Mechanistic work quantifying bacterial load and characterizing biofilm formation on different mesh chemistries could map the inflection point at which potentially septic becomes functionally septic for the mesh–peritoneum interface. Comparative experiments across mesh families (lightweight vs. heavyweight polypropylene, composite barriers with differing degradation kinetics) under identical contamination regimens would clarify whether any construct meaningfully widens the safety window in controlled contamination without sacrificing long-term stability. Extending follow-up beyond POD 8 would capture remodeling trajectories, testing whether early low-grade responses in clean/potentially septic settings remain benign or drift upward as collagen matures. Incorporating quantitative imaging and molecular readouts (e.g., cytokines, MMP/TIMP balance) alongside the current semi-quantitative histology could refine sensitivity to detect subclinical divergence that precedes overt adhesion formation. Together, such work would help translate these experimental insights into patient-level algorithms that balance recurrence prevention with infectious and adhesive risks in real-world contaminated fields.

In summary, the present data isolate sepsis—not merely the performance of a bowel anastomosis—as the pivotal driver of adverse interactions between intraperitoneal uncoated monofilament polypropylene mesh and the peritoneum in this experimental model. Clean and potentially septic conditions behaved comparably across inflammatory, fibroproliferative, angiogenic, collagen, and adhesion endpoints, whereas overt bacterial contamination produced a marked escalation in every domain examined. These findings provide mechanistic insight into the early biological response of this specific mesh construct under graded contamination conditions.

## 5. Conclusions

In this experimental model of graded intra-abdominal contamination, intraperitoneal polypropylene mesh demonstrated comparable early biological response patterns under clean and controlled contamination conditions, whereas higher degrees of intra-abdominal contamination were associated with increased local inflammatory and fibroproliferative activity and adhesion formation. These findings describe early postoperative tissue responses within a controlled experimental framework and should not be interpreted as evidence of long-term mesh behavior or direct clinical guidance. Further studies incorporating quantitative microbiological validation and extended follow-up are required to better characterize the durability and translational relevance of intraperitoneal polypropylene use in contaminated settings.

## Figures and Tables

**Table 1:** Histological score distribution by group and timepoint. Data represent the number of animals per semi-quantitative score (0–4). A = clean, B = anastomosis, C = controlled contamination (n = 10 per group per timepoint).

Parameter	POD	Group	0	1	2	3	4
Neutrophils	4	A	6	4	0	0	0
Neutrophils	4	B	4	4	2	0	0
Neutrophils	4	C	0	1	3	4	2
Neutrophils	8	A	5	5	0	0	0
Neutrophils	8	B	4	4	2	0	0
Neutrophils	8	C	0	0	3	4	3
Fibroblasts	4	A	7	3	0	0	0
Fibroblasts	4	B	5	4	1	0	0
Fibroblasts	4	C	0	2	4	4	0
Fibroblasts	8	A	6	4	0	0	0
Fibroblasts	8	B	4	4	2	0	0
Fibroblasts	8	C	0	0	4	5	1
Angiogenesis	4	A	8	2	0	0	0
Angiogenesis	4	B	5	5	0	0	0
Angiogenesis	4	C	0	2	5	3	0
Angiogenesis	8	A	7	3	0	0	0
Angiogenesis	8	B	5	4	1	0	0
Angiogenesis	8	C	0	0	5	5	0
Collagen	4	A	7	3	0	0	0
Collagen	4	B	5	5	0	0	0
Collagen	4	C	0	2	5	3	0
Collagen	8	A	8	2	0	0	0
Collagen	8	B	5	4	1	0	0
Collagen	8	C	0	1	5	4	0

## Data Availability

The dataset is available upon request from the authors.
